# Improving mammography access for women with disabilities: Outcomes of the CDC’s right to know campaign

**DOI:** 10.15761/fwh.1000188

**Published:** 2020-07-06

**Authors:** Meg A Traci, Holly Horan, Helen Russette, Rebecca Goe, Desirae Ware, Kim Powell, Rosemary B Hughes, Emily Hicks

**Affiliations:** 1 University of Montana Rural Institute: A Center for Excellence in Developmental Disabilities, USA; 2 Department of Anthropology, University of Alabama, USA; 3 University of Montana, School of Public and Community Health Sciences, USA; 4 Missoula Partnership Health Center, USA

**Keywords:** mammography, breast cancer, health equity, women

## Abstract

Women with disabilities share similar risks for breast cancer as other women yet experience a lack of access to cancer screening and are less likely to receive screening mammograms in accordance with recommended guidelines. The present study evaluated mammography centers across the state of Montana in response to the Centers for Disease Control and Prevention’s Right to Know campaign, which focused on addressing barriers to breast cancer screening. Mammography centers were originally evaluated in 2009 and were reassessed in 2011 and 2015 after being given action plans to address accessibility barriers. The current study examined changes in accessibility across time in four priority areas: 1) van and standard parking, 2) exterior and interior routes, 3) mammography rooms, and 4) restrooms. Results indicate all mammography centers had a least one mammography machine that lowered for patients in a seated position and that accessibility of the four priority areas improved over time; however, improvements were still needed to encourage health equity for women with disabilities.

## Introduction

The National Cancer Institute projected 276, 480 new breast cancer diagnoses and 42,170 deaths due to breast cancer in U.S. women in 2020 [1]. According to these estimates, breast cancer is the most frequently diagnosed cancer, excluding skin cancers, and it is the second leading cause of cancer and all-cause mortality in women [2]. Early detection and treatment of breast cancer are critical to survival, and regular mammograms are promoted as an effective breast cancer screening for early detection of tumors in breast tissue [3]. Current Centers for Disease Control and Prevention (CDC) recommendations are that women ages 40 to 49 consult their physicians about receiving a mammogram and that women ages 50 to 74 have a mammogram every two years [4].

Women with disabilities, who represent 25% of U.S. women [5], share similar risks for breast cancer as other women yet experience a lack of access to cancer screening and are less likely to receive screening mammograms in accordance with recommended guidelines [6–16]. Frequently encountering medical personnel who lack disability-related training and sensitivity [17–20], women with disabilities may also lack disability-specific health-related information and access to medical facilities and accessible medical equipment [9–26]. Moreover, women with disabilities are often diagnosed at later stages and experience higher rates of breast cancer death compared to women without disabilities. Indeed, this mortality disparity persists even when women are diagnosed at the same stage [27].

In response to these disparities, the CDC conducted research into the barriers to breast cancer screening among women with physical disabilities. Findings revealed few breast cancer awareness messages appropriately addressed the needs of women with disabilities [28]. As a result, the CDC developed the Right to Know campaign (RTK) to promote breast cancer screening awareness in women with physical disabilities and pilot tested it with positive results among centers for independent living in four U.S. cities. Effective RTK communication strategies included leveraging community events organized by disability advocates and partnering with local mammography centers identified as having accessible services.

Subsequently, the CDC funded the Montana Disability and Health Program (MTDH) and three other disability and health state-based public health programs to implement RTK. The MTDH is a partnership between the Montana Department of Public Health and Human Services and the University of Montana Rural Institute: A University Center on Disabilities. Montana RTK community partners also included Montana’s four centers for independent living (CILs), the Montana Department of Public Health and Human Services’ Cancer Control Program (MCCP), the Montana Cancer Coalition (MCC), and the Montana Affiliate of Susan G. Komen for the Cure [29].

Rural women with disabilities, who comprise more than 25% of all women with disabilities [21], have faced medical service shortages, high rates of poverty, uninsured and underinsured status, and lack of transportation [22–24]. Before implementing the RTK campaign, MTDH assessed the statewide capacity for delivering accessible breast cancer screening. In a rural state with seven Indian reservations and the state-recognized Little Shell Tribe of Chippewa Indians, it was essential to integrate this assessment with statewide efforts to improve healthcare access overall. RTK messaging promotes awareness of the opportunities to plan for an accessible mammogram when scheduling an appointment. The aim of this project was to develop that awareness with standard and socially valid information for center staff and for the public about what mammography centers had accessible screening and diagnostic equipment and what accessibility barriers to address by planning accommodations with the appointment (e.g., scheduling an exam room with accessible equipment, planning for more time in the exam room if the dressing rooms were not accessible, identifying the nearest ADA accessible bathroom in the facility if the one adjacent to the mammography center was not accessible, scheduling with staff who had person-specific or disability-related experience.) A secondary aim was to establish a baseline of access barriers and facilitators for state mammography centers and coordinate targeted technical assistance with partners to improve access to mammography with women with and without disabilities overtime. This latter aim was defined by goals and objectives in the state cancer control plan that supported the collaborative and sustained approach on the project.

To establish a baseline, MTDH supported CIL staff with expertise in the Americans with Disabilities Act (ADA), related standards, and resources to conduct accessibility evaluations as part of a complete, statewide assessment of mammography and breast cancer screening access. This is an ongoing effort, and centers are re-evaluated regularly. This paper describes results from the original MTDH accessibility evaluations conducted in 2009 (Phase I), as well as results from follow-up evaluations conducted in 2011 and 2015 (Phases II and III). Evaluating at multiple time points allows for changes across time regarding the accessibility of mammography centers to be documented.

## Method

The original project consisted of statewide mammography center assessments, and results of the assessment were used in the development of the Montana Mammography Directory that was published online in 2009 (Phase I). The assessments were also used to inform the development of action plans for centers needing accessibility improvements. In order to determine the effectiveness of the action plans, a follow-up assessment was conducted in 2011 (Phase II) that examined potential changes in disability access of the centers. An updated Montana Mammography Directory was published online as a result of new information regarding the accessibility of the centers. Action plans were again developed for centers needing disability access improvements. Follow-up assessments were again conducted in 2015 (Phase III) to determine current disability access, and the data was compiled into an updated Montana Mammography Directory published online. The mammography centers will continue to be evaluated and results from the evaluations will be published in online directories. Detailed information about the assessments, action plans, and directories can be found below. The project involved collaborations with four CILs, the MCC and its Early Detection Implementation Team, the MCCP and its regional contractors located at health departments in metropolitan statistical areas, the Montana Affiliate of Susan G. Komen for the Cure, and local mammography centers.

## Data collection

### Establishing a baseline of accessibility and access issues using on-site assessments

MTDH maintains a scope of work and supports training of CIL staff to conduct on-site accessibility and access assessments to establish a comprehensive baseline at each mammography center and to provide resources and on-site technical assistance. Trained staff at each CIL are known as *Accessibility Ambassadors*. They usually work in one of the main CIL offices located in Billings, Great Falls, Helena, or Missoula and travel the state which is covered by CIL service areas. Accessibility Ambassadors are trained to: (1) evaluate Montana’s capacity to provide mammograms to women with disabilities, (2) increase healthcare providers’ knowledge of the barrier’s women in this population encounter when accessing mammography, and (3) educate the public about the accessibility and importance of mammograms for all women. Accessibility Ambassadors attend a one-day training session that involved lectures, video presentations, discussion, and supervision in assessing a mammography center using the Massachusetts Facility Assessment Tool (MFAT) [30] adopted for use in Montana with additional elements of interest to partners organized in a second tool, the General Facility Assessment Instrument (GFAI).

### Phase I

Of the 41 Montana mammography centers recognized by the Food and Drug Administration in 2009, 40 participated in the original study. One center was not in operation at the time of the original assessment. Centers were sent an informational packet with an informed consent form (*This study was submitted to The University of Montana Institutional Review Board (IRB) and the Billings Area Indian Health Services IRB. The IRB determined that this project was not research. However, the MTDH staff still asked all participating mammography centers to sign a consent form*). As consent forms were returned, Accessibility Ambassadors contacted the centers to schedule an assessment. The sample included urban and rural mammography centers that served communities ranging in size from less than 300 to more than 200,000 residents, as well as a mobile mammography screening unit, and four mammography centers on three Indian reservations.

At the beginning of each assessment, Accessibility Ambassadors provided staff with educational materials about breast health care for women with disabilities. These materials included CDC RTK materials and a DVD with self-study materials-*Breast Health Access for Women with Disabilities (BHAWD): Training for the Mammography Technologist*, which offered a 1.0 continuing education (CE) credit 1.0. The CE credit was approved by the American Society of Radiologic Technologists [31].

The Accessibility Ambassadors assessed the centers in four priority areas: 1) accessible van and standard parking, 2) accessible interior and exterior routes, 3) accessible mammography rooms, and 4) accessible restrooms on the same floor of the mammography room. To meet MFAT standards (i.e., ADA and other architectural standards), a center had to meet accessibility requirements in all four areas. Elements are not present for evaluation and did not factor into compliance determination. As this element was not present, it did not contribute to their determination of overall compliance. Furthermore, if a center had multiple items for evaluation (e.g., multiple entrances), only one had to be accessible for the center to be listed as compliant.

The Phase I process of completing the MFAT and GFAI in all Montana mammography centers lasted one year. Results were recorded onsite by the Accessibility Ambassadors and then forwarded to MTDH staff to be entered into the Statistical Package for the Social Sciences software (SPSS). The results from the onsite assessments were disseminated in three ways: (1) action plans, (2) the online Montana Mammography Directory, and (3) the Right to Know Campaign.

### Materials: mammography center accessibility action plans and resource toolkit

Participating mammography centers needing improvements were sent an electronic and a paper copy action plan that (1) detailed recommended accessibility improvements identified by the onsite assessment, (2) explained what the center needed to do to comply with the ADA, and (3) suggested what tasks to complete to be compliant with prompts to assign a responsible party. All centers received a resource toolkit including breast health education materials designed for women with physical disabilities, and ADA education materials. All centers also were sent the CDC RTK Dissemination kits once they were published by CDC (approximately 8 to 12 months following the assessments).

### Montana mammography directory

MTDH staff and partners organized the data from the MFAT and GFAI into the 2009–2010 Montana Mammography Directory. This online directory, which outlined the contact and accessibility information and additional services of participating Montana centers, was made available electronically and was promoted on community partners’ websites and at conferences and community events. Partners adopted terminology and iconography developed for a state resource on breast health resources that was no longer being maintained or distributed. Initially, the directory included version of the icons that were accessible (i.e., included alt text descriptions). Later versions of the directory did not include the icons to promote usability with screen reader and other technology.

### Phases II and III. Updating assessments, action plans, and directories

To maintain the statewide evaluation of access barriers and facilitators to mammography and on-going capacity building supports, we coordinated three outreach activities with state partners in 2011 (Phase II) and 2015 (Phase III). Partners coordinated communications with mammography centers to promote awareness and participation in these activities. In these phases, we first identified new or significantly remodelled mammography centers for on-site evaluations to establish or re-establish their baseline on access issues and coordinated those on-site assessments with Accessibility Ambassadors. In Phase III, we added on-site assessments of six health care facilities that regularly hosted the state’s two mobile mammography units (five facilities hosted one mobile unit and one facility hosted the other mobile unit) and included that information the directory with information about the mobile units. Second, we collected updates from mammography centers already in the directory with a postcard that mammography centers completed and returned to MTDH staff with basic information on any changes to access issues made since the most recent assessment. Third, we used information collected with the postcard to schedule a telephone interview with mammography center administrative leads and MTDH staff to identify more detailed changes in access issues. Before the interview, mammography center staff were emailed or mailed copies of their action plans. The phone interviews were structured to review the action plans and identify updates made to the center facility and services. If a center was unavailable to provide information about disability access via a phone interview, they were also given the opportunity to report accessibility data via a mailed questionnaire. MTDH staff then made relevant updates to the mammography centers’ action plans and directory profile pages. Mammography center staff reviewed and approved their updated profile pages for the next version of the directory.

In 2011 (Phase II), 40 mammography centers were included in an updated Montana Mammography Directory that provided information about each center’s accessibility. In Phase III, an updated Montana Mammography Directory was published with current information regarding the accessibility of 53 mammography centers statewide. Each mammography directory contains information regarding services offered, costs of services, whether or not a referral is required, languages spoken at the center, whether or not Medicaid/Medicare is accepted, whether or not transportation to the center can be provided, and the accessibility of parking, routes, restrooms, and mammography rooms. Centers are continuing to be updated and future research can note additional changes in disability access observed in Montana mammography centers over time.

### Analysis plan

The present study sought to evaluate changes in the accessibility of mammography centers to women with disabilities in Montana from 2009 to 2015 (i.e., assessed in Phases I, II and III). Analyses include de-identified data and descriptive statistics noting the number of accessible features observed in mammography centers statewide. Additionally, while more in-depth information is provided in the Montana Mammography Directories, the present study focuses on observed changes in the following categories: 1) accessible van and standard parking, 2) accessible exterior and interior routes, 3) accessible mammography rooms, and 4) accessible restrooms on the same floor as mammography rooms.

## Results

In 2009 and 2011, the sample consisted of 40 mammography centers across the state of Montana. In Phase III, the sample consisted of 49 mammography centers statewide, as new centers and locations had been developed. In some centers, data regarding accessibility features was either not present or unable to be obtained. Table 1 depicts the total number of centers examined each year, as well as the number of centers that were able to be assessed for each accessibility feature corresponding to the evaluation year.

### Accessible parking

**Figure 1.**
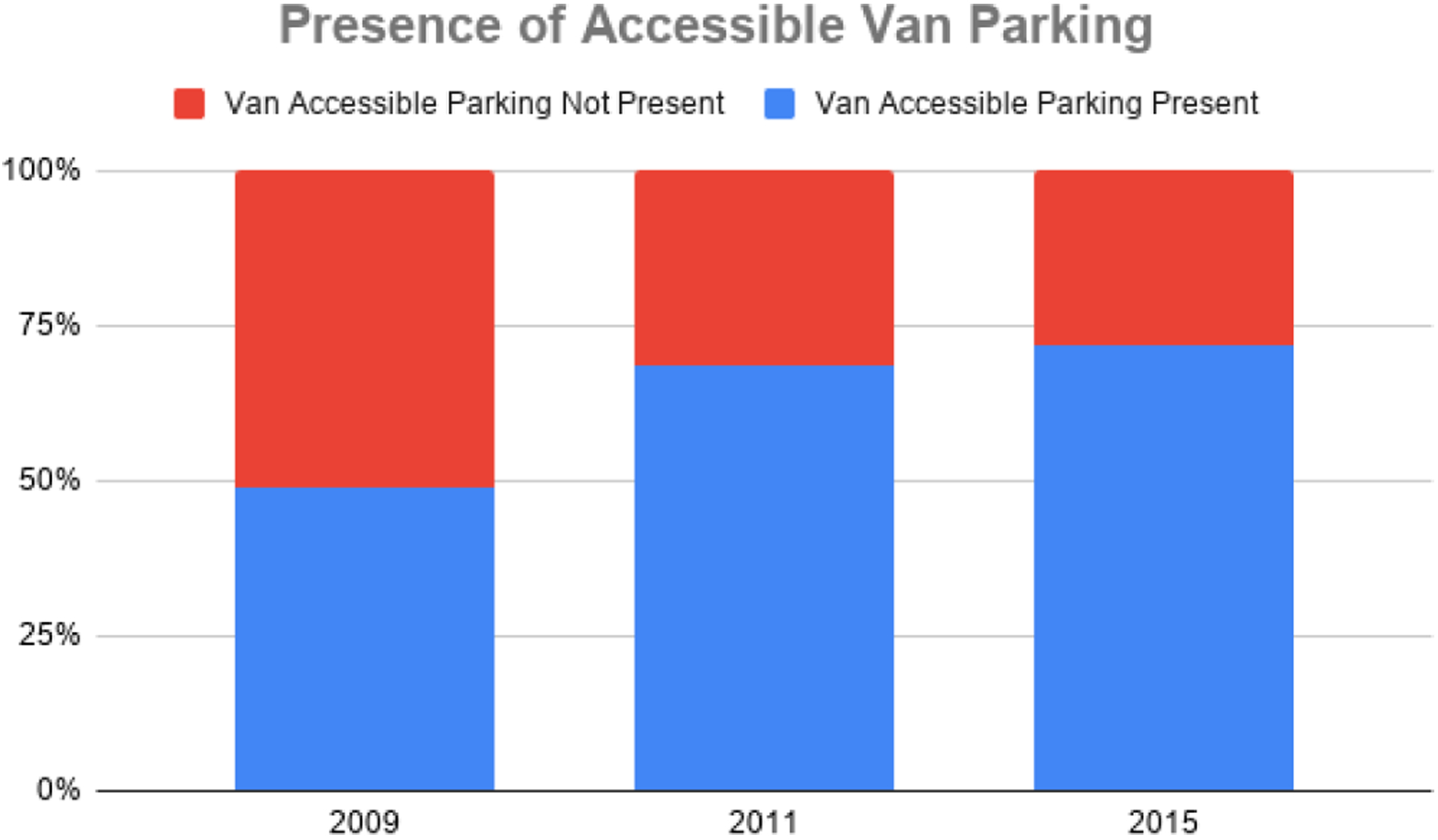
Presence of accessible van parking at Montana Mammography Centers (2009–2015).

**Figure 2.**
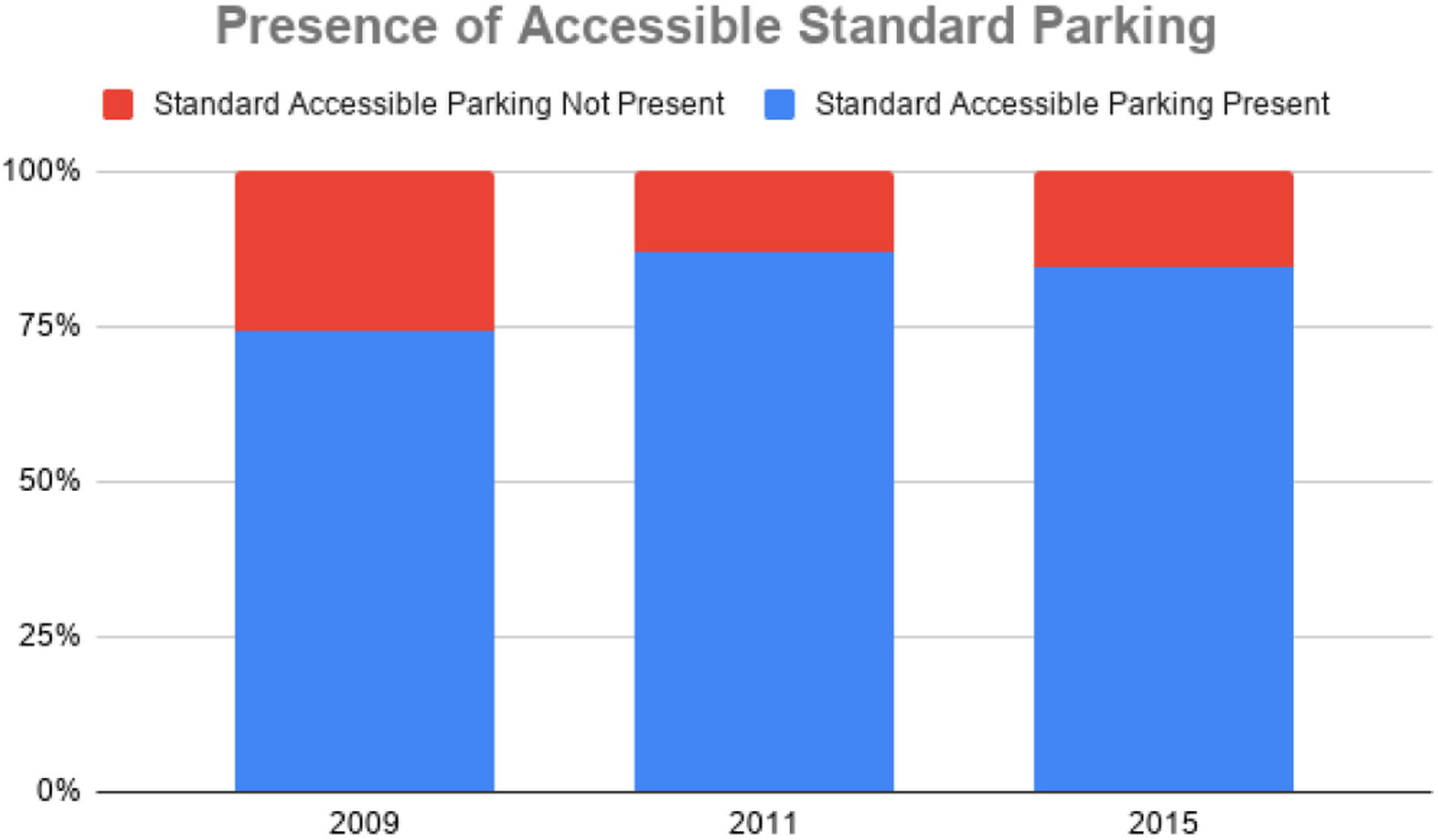
Presence of accessible standard parking at Montana Mammography Centers (2009–2015).

In 2009, about half of centers lacked accessible parking for vans (51.3%), and a quarter of centers lacked accessible standard parking (25.6%). When re-evaluated in 2011, after the action plans had been developed and disseminated, these numbers reduced to less than a third of centers lacking accessible van parking (31.6%) and an eighth of centers lacking accessible standard parking (12.8%). Centers were evaluated again in Phase III, and the results suggested that the percentage of centers lacking accessible parking for vans was reduced again to 28.2%, though the percentage of centers lacking accessible standard parking rose slightly to 15.4%. Overall, the findings indicate that the availability of accessible parking at mammography centers in Montana has increased over time. The presence of accessible van parking can be seen in figure 1, and the presence of accessible standard parking can be seen in figure 2.

### Accessible routes

**Figure 3.**
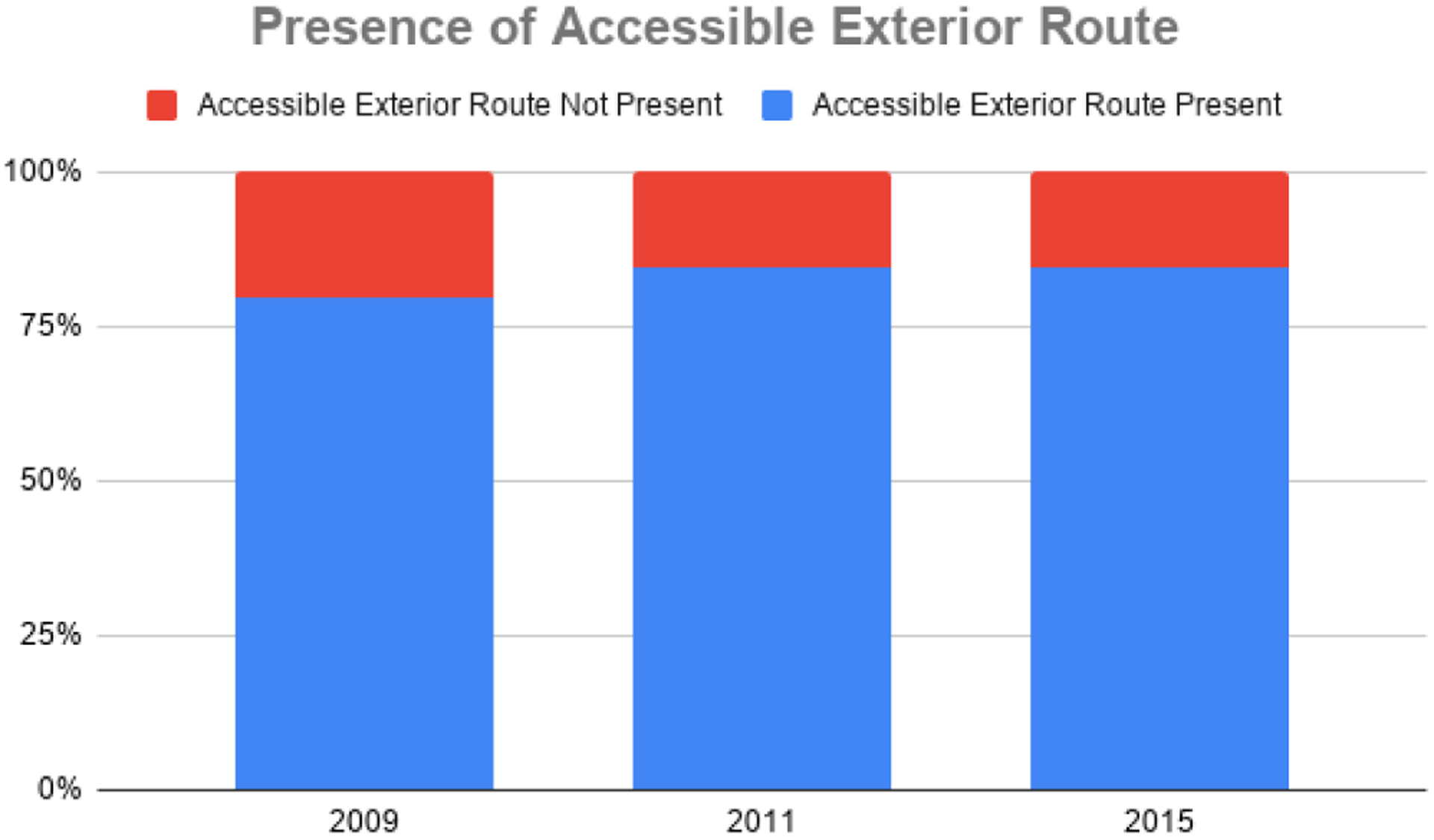
Presence of accessible exterior routes at Montana Mammography Centers (2009–2015).

**Figure 4.**
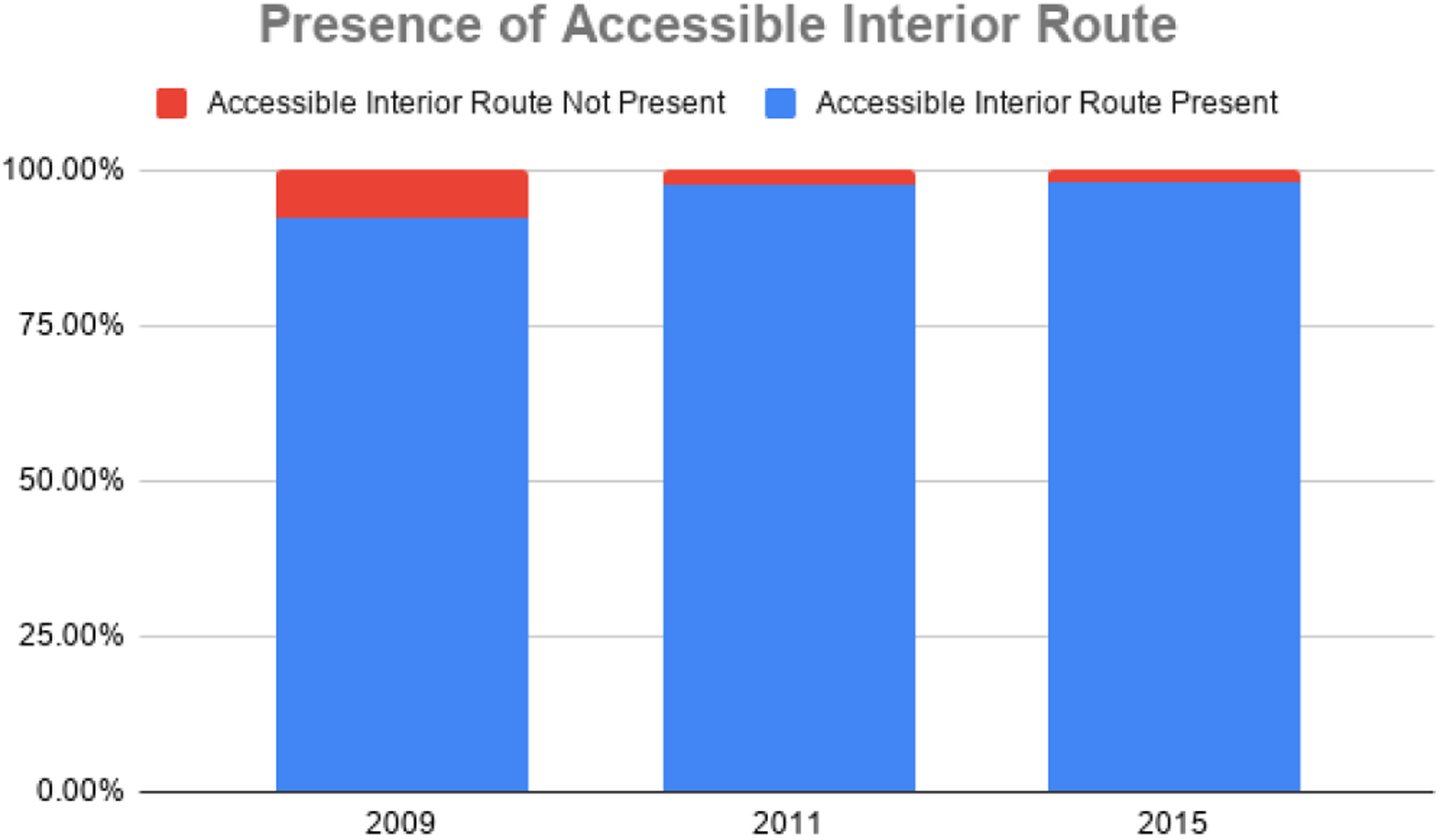
Presence of accessible interior routes at Montana Mammography Centers (2009–2015).

In 2009, one in five centers lacked accessible exterior routes (20.5%) and just three centers lacked accessible interior routes (7.5%). Results from the 2011 evaluation suggest that these numbers were reduced to 15.4% of centers lacking accessible exterior routes and 2.5% lacking accessible interior routes. After the Phase III assessment, the percentage of centers lacking accessible exterior routes remained the same at 15.4%, but the percentage of centers lacking accessible interior routes diminished to 2.1%. Overall, the results indicate that the availability of accessible exterior and interior routes in Montana mammography centers has improved over time. The presence of accessible exterior routes can be seen in figure 3, and the presence of accessible interior routes can be seen in figure 4.

### Accessible mammography rooms

**Figure 5.**
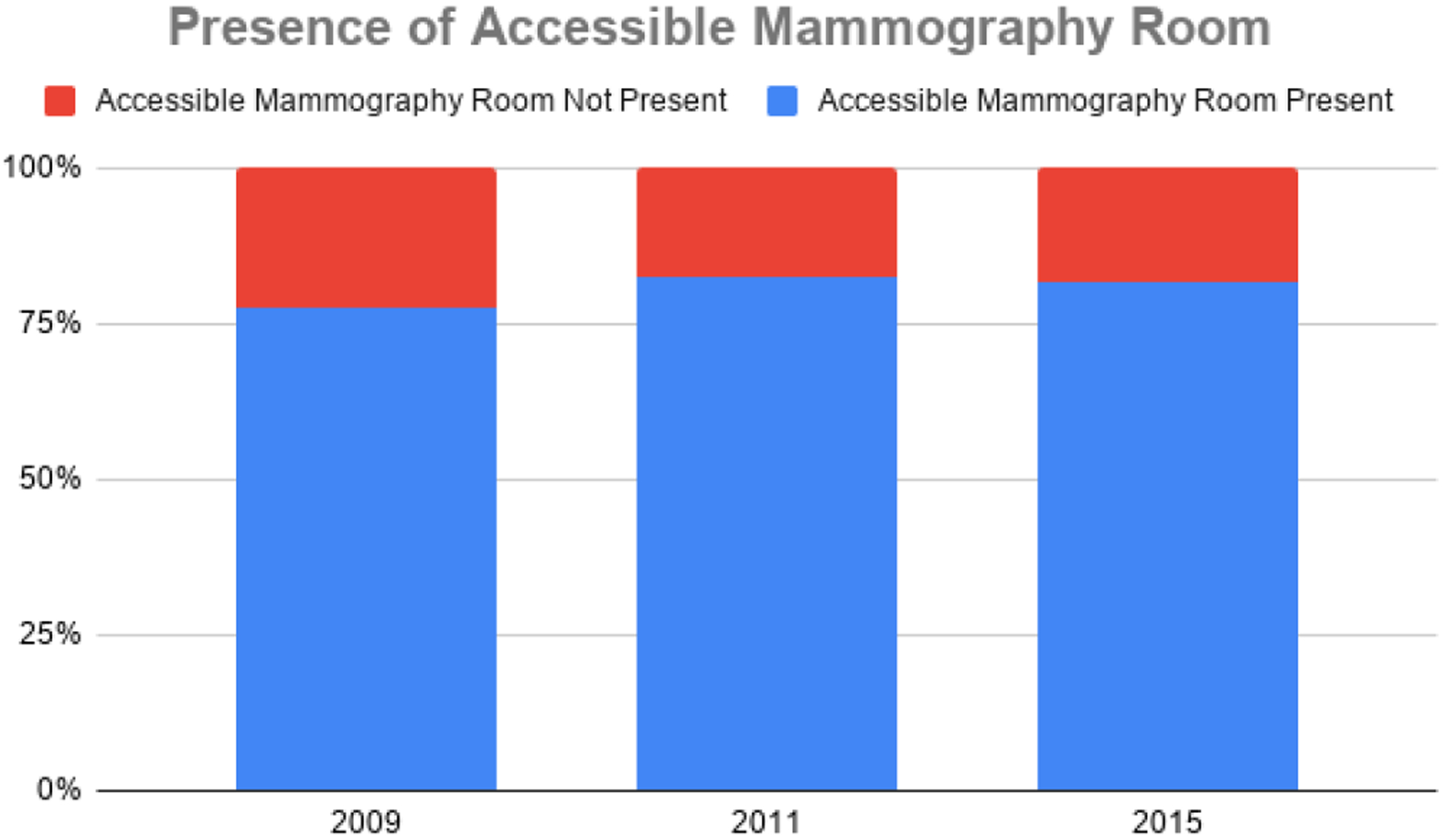
Presence of accessible mammography rooms at Montana Mammography Centers (2009–2015).

The 2009 evaluation of mammography centers indicated that about one in five centers lacked an accessible mammography room (22.5%). When assessed in 2011, after action plans had been distributed, this number was reduced to 17.5% of centers lacking an accessible mammography room. Results of the Phase III evaluations indicated that the percentage of centers without an accessible mammography room rose to 18.4%, a slight increase from the 2011 evaluations. The findings indicated that the availability of accessible mammography rooms has improved since the initial evaluation; however, there is still a need for greater accessibility in centers. This may be explained, in part by the accessibility issues associated with one of the mobile units used at several of the host facilities added in Phase III to the database and directory. Figure 5 depicts changes in the presence of the accessibility of mammography rooms over time.

### Accessible restrooms on the same floor

**Figure 6.**
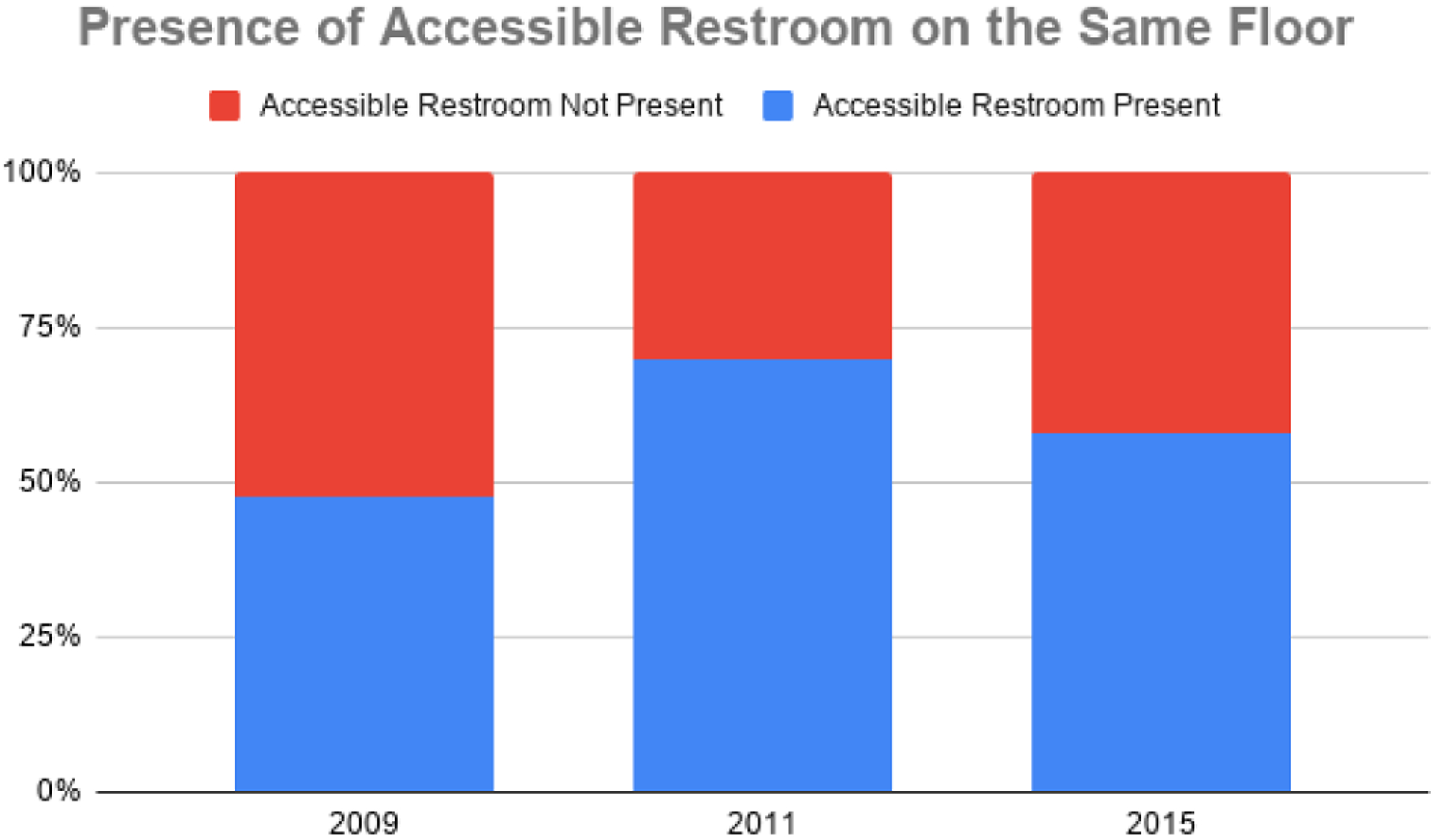
Presence of accessible restroom on same floor as Montana Mammography Centers (2009–2015).

In 2009, about half of centers did not have an accessible restroom available on the same floor as mammography rooms (52.5%). Results of the 2011 evaluation suggest that this number was reduced to less than one third of centers lacking an accessible restroom on the same floor (30.0%). In Phase III, more than two in five mammography centers lacked an accessible restroom on the same floor (42.2%). Out of all disability access features (parking, routes, mammography rooms, and restrooms), accessible restrooms on the same floor as mammography rooms continued to be the feature with the least accessibility. This may be explained in part, by the inclusion of facilities hosting the mobile units having older infrastructure, including lack of an accessible bathroom. The presence of accessible restrooms in Phase I, Phase II, and Phase III is depicted in figure 6.

### Total accessibility

**Figure 7.**
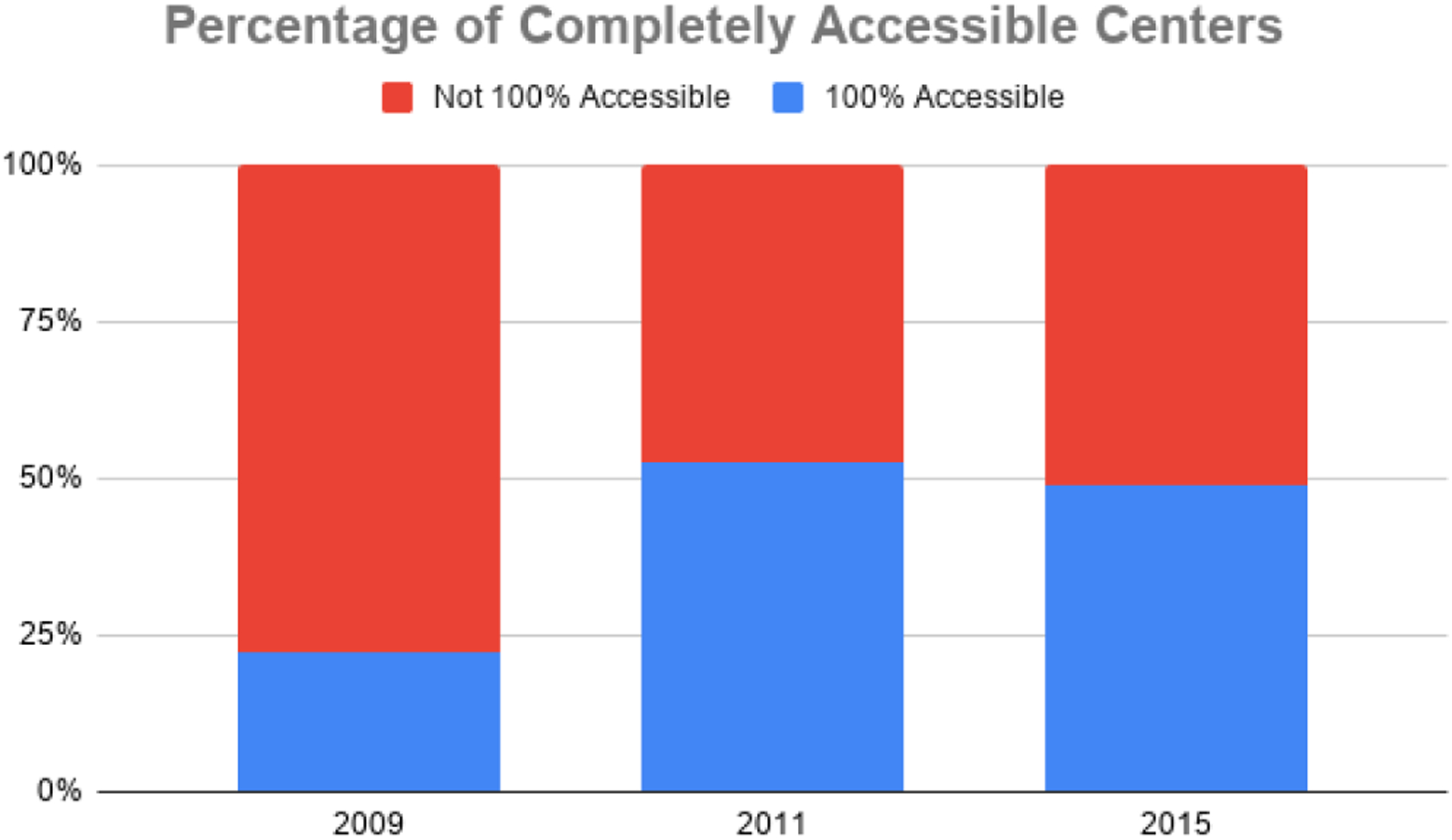
Presence of completely accessible Montana Mammography Centers (2009–2015).

In order to be considered totally accessible, a center had to meet criteria for accessibility in all four priority areas. In 2009, only 22.5% of centers across the state met the requirements to be considered completely accessible. This percentage was raised after the 2011 evaluations to 52.5%. Findings from the Phase III assessments indicate that this number decreased slightly and 49.0% of centers statewide met criteria to be considered totally accessible. This may be explained in part, by the inclusion of rural facilities hosting the mobile units having older infrastructure. Figure 7 depicts the presence of completely accessible mammography centers across time.

## Discussion

Overall, centers have shown a low level of compliance with ADA guidelines with approximately half of centers across the state failing to meet accessibility criteria in the four priority areas in Phase III. The lack of compliance has indicated the need for continued education and advocacy concerning ADA standards in medical centers, especially as equipment is replaced and new buildings are constructed.

Despite the overall lack of compliance with ADA guidelines, improvements have continuously been seen with regard to the accessibility of mammography centers van and standard parking, exterior and interior routes, mammography rooms, and restrooms. Results of the evaluations suggest that Montana mammography centers have increased disability access, though improvements are still needed. With continued evaluations and action plans, the accessibility of mammography centers may continue to increase.

To determine the capacity of preventive and primary healthcare systems to serve the entire public, assessments must include accessibility as a core component. Without accessibility data, improvement plans may omit critical actions needed to ensure equitable health care access for populations with disabilities. The ADA has provided excellent guidelines for feasible accessibility options. A public health approach to these barriers will unveil further features of accessibility that can be used to make it a standard component of care for patients.

Socioeconomic status, disability and health insurance can all contribute to health outcomes in people with disabilities, but other barriers to healthcare also exist. Environmental factors such as climate, terrain, social attitudes, institutions and laws can also affect disability and functioning [32]. Medical facilities may be some of the oldest local institutions and can present issues with the built environment that include inaccessible entrances, causing people using wheelchairs to enter the building through other means (i.e., loading docks), as well as inaccessible equipment, restrooms, and elevators [33].Continually facing these obstacles, inconveniences, and dangers is exhausting and demoralizing for women with disabilities [33–35].While the accessibility of Montana mammography centers has improved, centers should be encouraged to continue making the environment more inclusive and accessible for all people in order to improve patient care.

In terms of the RTK campaign, all centers did have a least one mammography machine that lowered to a low seated position as assessed by a trusted member of the disability community. This meant that we could promote screening guidelines to women with physical disabilities with the campaign’s patient advocacy tips designed for women with disabilities to coordinate needed accommodations and supports for accessing that piece of equipment. Mammography centers reported anecdotally that the project helped them understand their accessible equipment and features as assets to protect and grow with opportunities to purchase new equipment and remodel their facilities. Mammography centers also increased their support in implementing the RTK campaign and partnered with MTDH and Montana CILs to co-host community events that launched the campaign in twelve counties and at state and regional events. We continue to work with our partners promote greater awareness of the U.S. Access Board standards for *Accessible Medical Diagnostic Equipment* and opportunities for continuing education and other capacity building activities.

## Limitations

Some mammography facility managers were unaware of the accessibility assessment and the action plans that accompanied them, indicating the need to continue outreach efforts with Montana centers. Documents can be misplaced during administrative shifts, or in a facility with limited supervisory staff, the information may be delegated to other center staff. Creating a heightened awareness, in all center staff, concerning the social and emotional experience of mammograms for women with disabilities may be useful in future applications of mammography facility assessments.

The Montana Mammography Directories contain information regarding services offered, cost of services, and whether or not Medicaid / Medicare is accepted at the center. The present study did not evaluate this data, but future research can determine the feasibility of receiving services at various centers by including this information.

## Conclusion

In Montana, barriers for women with disabilities have been reduced or eliminated as mammography centers make necessary upgrades to their facilities to provide equal access. However, problems have remained due to the lack of physical access to buildings and, once inside, access to resources that need to be available and accessible for patients. We have not determined if these improvements resulted from the RTK campaign and this project or from independent construction plans, or a combination of these factors. Regardless, these improvements will meet the increasing demand for accessible breast cancer screening.

## Figures and Tables

**Table 1.** Number of centers assessed by feature per year

Accessibility Feature	2009	2011	2015
Van Parking	39	38	39
Standard Parking	39	39	39
Exterior Route	39	39	39
Interior Route	40	40	48
Mammography Room	40	40	49
Restroom	40	40	45
**Total N of Centers Assessed**	40	40	49
